# National Evaluation of the Association Between Resident Labor Union Participation and Surgical Resident Well-being

**DOI:** 10.1001/jamanetworkopen.2021.23412

**Published:** 2021-09-01

**Authors:** Brian C. Brajcich, Jeanette W. Chung, Douglas E. Wood, Karen D. Horvath, Philip D. Tolley, Elizabeth F. Yates, Chandrakanath Are, Ryan J. Ellis, Yue-Yung Hu, Karl Y. Bilimoria

**Affiliations:** 1 Surgical Outcomes and Quality Improvement Center, Department of Surgery, Northwestern Medicine, Chicago, Illinois; 2 Department of Surgery, UW Medicine, Seattle, Washington; 3 Department of Surgery, Brigham Health, Boston, Massachusetts; 4 Department of Surgery, University of Nebraska Medical Center, Omaha

## Abstract

**Importance:**

Labor unions are purported to improve working conditions; however, little evidence exists regarding the effect of resident physician unions.

**Objective:**

To evaluate the association of resident unions with well-being, educational environment, salary, and benefits among surgical residents in the US.

**Design, Setting, and Participants:**

This national cross-sectional survey study was based on a survey administered in January 2019 after the American Board of Surgery In-Training Examination (ABSITE). Clinically active residents at all nonmilitary US general surgery residency programs accredited by the American Council of Graduate Medical Education who completed the 2019 ABSITE were eligible for participation. Data were analyzed from December 5, 2020, to March 16, 2021.

**Exposures:**

Presence of a general surgery resident labor union. Rates of labor union coverage among non–health care employees within a region were used as an instrumental variable (IV) for the presence of a labor union at a residency program.

**Main Outcomes and Measures:**

The primary outcome was burnout, which was assessed using a modified version of the abbreviated Maslach Burnout Inventory and was defined as experiencing any symptom of depersonalization or emotional exhaustion at least weekly. Secondary outcomes included suicidality, measures of job satisfaction, duty hour violations, mistreatment, educational environment, salary, and benefits.

**Results:**

A total of 5701 residents at 285 programs completed the pertinent survey questions (response rate, 85.6%), of whom 3219 (56.5%) were male, 3779 (66.3%) were White individuals, 449 (7.9%) were of Hispanic ethnicity, 4239 (74.4%) were married or in a relationship, and 1304 (22.9%) had or were expecting children. Among respondents, 690 residents were from 30 unionized programs (10.5% of programs). There was no difference in burnout for residents at unionized vs nonunionized programs (297 [43.0%] vs 2175 [43.4%]; odds ratio [OR], 0.92 [95% CI, 0.75-1.13]; IV difference in probability, 0.15 [95% CI, −0.11 to 0.42]). There were no significant differences in suicidality, job satisfaction, duty hour violations, mistreatment, educational environment, salary, or benefits except that unionized programs more frequently offered 4 weeks instead of 2 to 3 weeks of vacation (27 [93.1%] vs 52 [30.6%]; OR, 19.18 [95% CI, 3.92-93.81]; IV difference in probability, 0.77 [95% CI, 0.09-1.45]) and more frequently offered housing stipends (10 [38.5%] vs 9 [16.1%]; OR, 2.15 [95% CI, 0.58-7.95]; IV difference in probability, 0.62 [95% CI 0.04-1.20]).

**Conclusions and Relevance:**

In this evaluation of surgical residency programs in the US, unionized programs offered improved vacation and housing stipend benefits, but resident unions were not associated with improved burnout, suicidality, job satisfaction, duty hour violations, mistreatment, educational environment, or salary.

## Key Points

**Question:**

Is the presence of a resident labor union associated with improved well-being at US surgical training programs?

**Findings:**

In this cross-sectional survey study of 5701 residents, unionized programs were more likely to offer housing stipends and 4 weeks (instead of 2-3 weeks) of vacation time to residents; however, no difference in burnout, suicidality, job satisfaction, duty hour violations, mistreatment, salary, or the educational environment were found between residents at unionized and nonunionized programs.

**Meaning:**

These findings suggest that resident labor unions do not appear to improve resident well-being.

## Teaser

This survey study evaluates the association of resident physician unions with burnout, suicidality, job satisfaction, duty hour violations, mistreatment, educational environment, salary, and benefits among surgical residents in the US.

## Introduction

Studies show that burnout is common among health care professionals.^1,2^ Burnout is characterized by emotional exhaustion, depersonalization, and low personal accomplishment^3^ and is associated with medical errors, suicide, attrition, and substance abuse.^4,5,6^ Trainees, particularly in surgery, are at heightened risk for burnout, duty hour violations, and mistreatment.^1,2,7,8,9^ Mistreatment, long hours, poor compensation, and job-related stress may contribute to resident physician burnout.^2,10,11^

Through collective bargaining and advocacy, labor unions can improve working conditions and employee benefits^12,13^ and have been advocated as a means of improving resident well-being.^14,15,16,17^ However, concerns have been raised that resident unions may reframe the student-educator relationship as adversarial, introduce nonmedical arbiters into decisions about clinical duties, and harm professionalism.^15,18^ Unionization opponents contend that other means, including advocacy through institutional house staff associations or the American Council on Graduate Medical Education (ACGME), may be better avenues of affecting change.^18^

Discussions regarding unionization have intensified owing to the additional challenges faced by residents during the COVID-19 pandemic.^15^ However, no recent data exist regarding the effect of unions on resident-reported outcomes. Therefore, the objective of this study was to undertake a national evaluation of the association of resident unions with burnout, suicidality, job satisfaction, duty hour violations, mistreatment, educational environment, salary, and benefits in a population that is at particularly high risk for burnout and mistreatment. To our knowledge, this is the first US study to evaluate the association of resident unions with well-being and working conditions among residents.

## Methods

### Study Setting and Participants

In this cross-sectional survey study, a survey was administered to all general surgery residents who completed the 2019 American Board of Surgery In-Training Examination (ABSITE), a computer-based examination taken annually by residents at ACGME-accredited general surgical residency programs. The survey was administered immediately after the ABSITE in January 2019 using the examination software. A statement preceding the survey informed respondents that the survey was research related, data would be deidentified, and individual responses would not be provided to department or residency leadership. There were no incentives or disincentives to participate. Any examinee who initiated the survey was considered a respondent, and response rates were calculated according to the response rate 2 definition from the American Association for Public Opinion Research (eFigure in the Supplement).^19^ The study population included clinically active US surgical residents who completed the 2019 ABSITE. Military programs (n = 9) were excluded because members of the armed services are not permitted to unionize; programs in Puerto Rico (n = 2) were excluded because regional labor data were not available; and programs that closed (n = 4) after the 2019 ABSITE were excluded. Because the data were deidentified, the Northwestern University Institutional Review Board deemed this study exempt from human subjects research review. Informed consent was waived, but survey completion was voluntary. This study followed the American Association for Public Opinion Research (AAPOR) reporting guideline.

### Survey Development

The 2019 survey was adapted from previous surveys administered following the ABSITE.^2,20^ Surveys were based on validated instruments in the literature.^21,22,23,24,25^ To assess overall survey coherence, balance, and clarity, pretest cognitive interviews were conducted with general surgery residents from multiple institutions; based on this feedback, the survey was iteratively revised. The survey is available in eMethods 1 in the Supplement.

### Resident and Program Characteristics

Demographic factors including race, ethnicity, relationship status, and parental status were reported by survey respondents; respondents could select 1 or more options for their race. Resident and program characteristics provided by the American Board of Surgery included sex, postgraduate year, program type, geographic region, and program size. Urban-rural classification was obtained from the 2013 National Center for Health Statistics Urban-Rural Classification Scheme for Counties.^26^

Unionization information was obtained via a survey of residency program directors and coordinators, program website review, and examination of the member institution list on the Committee of Interns and Residents/Service Employees International Union (CIR/SEIU) website.^14^ Programs were classified based on whether residents were members of a labor union at the time of the 2019 ABSITE. For unionized programs, time since unionization and affiliation with a national union (eg, CIR/SEIU) were collected.

### Outcomes

The primary outcome was burnout, assessed by a modified version of the abbreviated Maslach Burnout Inventory–Human Services Survey for Medical Personnel (aMBI).^2,10,27^ This validated instrument consists of 3 domains: depersonalization, emotional exhaustion, and personal accomplishment. Each domain is assessed by 3 questions, with responses following a 7-option Likert scale (never, a few times a year, once a month or less, once a week, a few times a week, or every day). Burnout was defined as experiencing any depersonalization or emotional exhaustion symptom at least weekly, consistent with prior literature^1,2,5,28,29,30^ that has demonstrated the validity of the aMBI, the use of a 2-domain definition, and a threshold of experiencing symptoms weekly.

Resident-reported secondary outcomes included measures of suicidality, job satisfaction, duty hour violations (working more than 80 h/wk, receiving less than 1 in 7 days off, or taking in-house call more than 1 in 3 days), mistreatment (discrimination due to sex, gender identity, sexual orientation, race, ethnicity, or religion; bullying; or sexual harassment), and educational environment. Likert scale responses were categorized as strongly agree or agree vs neutral, disagree, or strongly disagree, as in prior work.^1,2^ Program-level secondary outcomes included salary, vacation duration, and whether subsidized childcare or housing, relocation, or technology stipends were provided. These data were obtained from the American Medical Association FREIDA website,^31^ review of program websites, and publicly available employment contracts. Salary was standardized to the 2020-2021 academic year; if data were only available for another year, the 2020-2021 salary was estimated based on a 3% annual increase.^32^

### Statistical Analyses

Data were analyzed from December 5, 2020, to March 16, 2021. The association of unions with outcomes was assessed using logistic regression for dichotomous outcomes and linear regression for continuous outcomes. A separate model was constructed for each outcome. Models of resident-reported outcomes included sex, race, Hispanic ethnicity, program type, census region, urban-rural classification, and program size as covariates and used cluster-robust standard errors. Models of program outcomes included program type, census region, urban-rural classification, and program size. Salary models also included county median household income, and housing stipend models also included county median rent, both obtained from the 2013-2017 US Census Bureau American Community Survey Summary File.^33^ A stratified analysis was performed for programs within and outside the New York–Newark combined statistical area (CSA). This analysis was performed for 2 reasons. First, by separately studying residents at programs within an urban area, any unmeasured confounding related to geographic location can be eliminated. Second, New York has a unique history related to resident duty hour reform and has a disproportionate number of unionized programs. Owing to the unique nature of medical education in New York, the region was studied separately to explore the generalizability of study findings.

To address unmeasured confounders and the possibility that residents unionized in response to poor working conditions, instrumental variable (IV) methods were also used. Instrumental variable analysis is an econometric technique that estimates the causal association of an exposure and outcome in the presence of other associations, such as unmeasured confounders or reverse causality.^34^ This analysis is accomplished through pseudorandomization using an IV: a variable strongly associated with the exposure, but which leads to the outcome only through the studied exposure. Instrumental variable techniques were used in this study to address 2 limitations of traditional regression techniques: (1) the presence of unmeasured confounders (eg, program-specific culture) and (2) the possibility that unions formed because of poor working conditions (eMethods 2 in the Supplement).

The regional unionization rate of non–health care public sector employees was used as an IV for residency program unionization. This rate was calculated from the August 2015 through October 2020 US Census Bureau Current Population Survey as the fraction of non–self-employed civilians 16 years or older who were employed full-time in non–health care occupations in the public sector and reported being covered by a labor union.^35,36^ Unionization rates were calculated within each CSA, within each metropolitan statistical area, or across nonmetropolitan regions of a given state. Instrumental variable regression models were constructed using the 2-stage least-squares method as linear probability models for dichotomous outcomes and linear models for continuous outcomes using the same covariates as the naive models. First-stage F statistics are reported in eTable 1 in the Supplement.

Several sensitivity analyses were performed and are presented in eTables 3 to 5 in the Supplement. First, burnout was modeled as a continuous variable, which was calculated as the sum of the scores for all items in the emotional exhaustion and depersonalization domains (range, 0 [never] to 6 [every day]). Second, because the effect of unionization may not be immediate, programs unionized for more than 3 years were compared with those that were not unionized or were unionized for less than 3 years. Finally, all dichotomous outcomes were modeled using linear probability models to permit side-by-side comparison of naive and IV model estimates. All tests of statistical significance were 2 sided, and a threshold of *P* < .05 was used. Significance is reported with and without Bonferroni adjustment. Statistical analyses were performed using SAS, version 9.4 (SAS Institute Inc) and STATA, version 16.0 (StataCorp LLC).

## Results

In total, 6661 residents at 285 eligible programs responded to the survey (response rate, 85.6%). Among 5701 residents who completed the aMBI, 3219 (56.5%) were male, 2339 (41.0%) were female, and 143 (2.5%) were missing information regarding their sex; 3779 (66.3%) were White individuals; 449 (7.9%) were of Hispanic ethnicity; 4239 (74.4%) were married or in a relationship; and 1304 (22.9%) had or were expecting children. A total of 690 residents at 30 programs (10.5% of programs) were unionized, and 5011 residents at 255 programs (89.5% of programs) were not. Of the 30 unionized programs, 25 (83.3%) were CIR/SEIU affiliated, 3 (10.0%) were independent, and 2 (6.7%) were affiliated with other unions. Twenty-six programs (86.7%) had been unionized for more than 3 years. Additional study population characteristics are presented in Table 1, and characteristics of survey respondents and nonrespondents are shown in eTable 2 in the Supplement.

**Table 1.** Characteristics of Residents From 285 Surgical Residency Programs

Characteristic	Study group^a^		
	Overall	Unionized	Nonunionized
**Residents **				
No. of participants	5701	690	5011
Sex			
Male	3219 (56.5)	383 (55.5)	2836 (56.6)
Female	2339 (41.0)	270 (39.1)	2069 (41.3)
Missing	143 (2.5)	37 (5.4)	106 (2.1)
Race^b^			
White	3779 (66.3)	356 (51.6)	3423 (68.3)
Black	283 (5.0)	50 (7.2)	233 (4.6)
Asian	1008 (17.7)	161 (23.3)	847 (16.9)
Other^c^	577 (10.1)	109 (15.8)	468 (9.3)
Prefer not to say	281 (4.9)	40 (5.8)	241 (4.8)
Hispanic ethnicity			
Yes	449 (7.9)	79 (11.4)	370 (7.4)
No	4886 (85.7)	553 (80.1)	4333 (86.5)
Prefer not to say or missing	366 (6.4)	58 (8.4)	308 (6.1)
Relationship status			
Married or in a relationship	4239 (74.4)	495 (71.7)	3744 (74.7)
Single, divorced, or widowed	1437 (25.2)	188 (27.2)	1249 (24.9)
Missing	25 (0.4)	7 (1.0)	18 (0.4)
Have or expecting children			
Yes	1304 (22.9)	139 (20.1)	1165 (23.2)
No	4350 (76.3)	540 (78.3)	3810 (76.0)
Missing	47 (0.8)	11 (1.6)	36 (0.7)
Year of training			
PGY-1	1377 (24.2)	180 (26.1)	1197 (23.9)
PGY-2 or PGY-3	2295 (40.3)	270 (39.1)	2025 (40.4)
PGY-4 or PGY-5	2029 (35.6)	240 (34.8)	1789 (35.7)
**Program**				
No. of programs	285	30	255
Program type			
Academic	133 (46.7)	16 (53.3)	117 (45.9)
Community	152 (53.3)	14 (46.7)	138 (54.1)
Program size, median (IQR), No. of examinees	25 (18-40)	35 (20-49)	25 (17-39)
Census region			
Northeast	85 (29.8)	18 (60.0)	67 (26.3)
South	87 (30.5)	2 (6.7)	85 (33.3)
Midwest	73 (25.6)	1 (3.3)	72 (28.2)
West	40 (14.0)	9 (30.0)	31 (12.2)
Urban-rural classification^d^			
Large metropolitan core	142 (49.8)	25 (83.3)	117 (45.9)
Large metropolitan fringe	40 (14.0)	3 (10.0)	37 (14.5)
Small or medium metropolitan or micropolitan	103 (36.1)	2 (6.7)	101 (39.6)
Faculty wellness champion^e^	94 (46.5)	11 (52.4)	83 (45.9)
Union affiliation			
CIR/SEIU	NA	25 (83.3)	NA
Independent	NA	3 (10.0)	NA
Other	NA	2 (6.7)	NA
Time since union creation, y			
1-3	NA	4 (13.3)	NA
>3	NA	26 (86.7)	NA

Abbreviations: CIR/SEIU, Committee of Interns and Residents/Service Employees International Union; IQR, interquartile range; NA, not applicable; PGY, postgraduate year.

^a^ Unless otherwise indicated, data are expressed as number (%) of residents or programs. Percentages have been rounded and may not total 100.

^b^ Residents were permitted to select more than 1 race. Fourteen residents were missing responses for all race variables. Residents with missing responses for each race variable were included in analyses with missing data treated as a separate response level.

^c^ Includes residents who indicated American Indian or Alaska Native, Native Hawaiian or Other Pacific Islander, or other.

^d^ National Center for Health Statistics urban-rural classification of the county containing each program. No residency programs were in rural noncore counties unassociated with a metropolitan or micropolitan core.

^e^ Data reported for the 202 residency programs (21 unionized and 181 nonunionized) that provided a response to this survey item.

### Burnout, Suicidality, Job Satisfaction, Duty Hour Violations, and Mistreatment

Overall, 2472 residents (43.4%) reported experiencing 1 or more burnout symptoms at least weekly. Burnout did not differ between residents at unionized vs nonunionized programs (297 of 690 [43.0%] vs 2175 of 5011 [43.4%]) by logistic regression (odds ratio [OR], 0.92 [95% CI, 0.75-1.13]) or IV analysis (difference in probability, 0.15 [95% CI, −0.11 to 0.42]) (Table 2). On naive and IV regression analysis, no significant difference was observed between residents at unionized vs nonunionized programs for suicidality (26 of 690 [3.8%] vs 234 of 5011 [4.7%]), thoughts of attrition (89 of 689 [12.9%] vs 578 of 4996 [11.6%]), dissatisfaction with the decision to become a surgeon (37 of 685 [5.4%] vs 253 of 5004 [5.1%]), or dissatisfaction with time for rest (152 of 688 [22.1%] vs 897 of 5004 [17.9%]). No differences were seen in duty hour violations (299 of 672 [44.5%] vs 2079 of 4911 [42.3%]), discrimination (344 of 630 [54.6%] vs 2490 of 4657 [53.5%]), or bullying (441 of 668 [66.0%] vs 3294 of 4906 [67.1%]). Reported rates of sexual harassment were significantly lower at unionized programs (170 of 668 [25.5%] vs 1525 of 4868 [31.3%]; OR, 0.70 [95% CI, 0.56-0.87]); however, this rate did not persist on IV analysis (difference in probability, −0.07 [95% CI, −0.27 to 0.12]).

**Table 2.** Association Between Program Unionization Status and Resident Outcomes^a^

Outcome	Resident group, No./total No. (%)		Logistic regression, OR (95% CI)^b^	IV analysis, difference in probability (95% CI)^c^
	Unionized	Nonunionized		
Burnout	297/690 (43.0)	2175/5011 (43.4)	0.92 (0.75 to 1.13)	0.15 (−0.11 to 0.42)
Suicidal ideation	26/690 (3.8)	234/5011 (4.7)	0.69 (0.44 to 1.08)	−0.08 (−0.17 to 0.01)
Job satisfaction				
Thoughts of attrition	89/689 (12.9)	578/4996 (11.6)	1.00 (0.76 to 1.31)	0.08 (−0.09 to 0.24)
Dissatisfied with decision to become a surgeon	37/685 (5.4)	253/5004 (5.1)	1.02 (0.66 to 1.56)	0.11 (0.00 to 0.23)
Dissatisfied with time for rest	152/688 (22.1)	897/5004 (17.9)	1.01 (0.74 to 1.38)	−0.07 (−0.28 to 0.13)
Duty hour violations	299/672 (44.5)	2079/4911 (42.3)	0.88 (0.65 to 1.18)	−0.30 (−0.65 to 0.05)
Mistreatment				
Any discrimination^d^	344/630 (54.6)	2490/4657 (53.5)	0.88 (0.70 to 1.09)	−0.05 (−0.23 to 0.13)
Bullying	441/668 (66.0)	3294/4906 (67.1)	0.85 (0.70 to 1.05)	0.05 (−0.23 to 0.33)
Sexual harassment	170/668 (25.5)	1525/4868 (31.3)	0.70 (0.56 to 0.87)^e^	−0.07 (−0.27 to 0.12)
Educational environment				
Dissatisfied with educational quality	105/688 (15.3)	456/5003 (9.1)	1.49 (1.03 to 2.17)	0.05 (−0.14 to 0.24)
Inadequate time for patient care	99/686 (14.4)	458/4982 (9.2)	1.38 (1.01 to 1.90)	−0.02 (−0.17 to 0.12)
Lack of protected educational time	124/678 (18.3)	610/4959 (12.3)	1.47 (0.95 to 2.29)	0.00 (−0.23 to 0.23)
Inadequate time in operating room	93/681 (13.7)	363/4957 (7.3)	1.55 (0.97 to 2.49)	0.00 (−0.15 to 0.16)
Inadequate autonomy in operating room	83/682 (12.2)	427/4955 (8.6)	1.14 (0.72 to 1.82)	0.00 (−0.20 to 0.20)
Inadequate autonomy in clinical decisions	37/682 (5.4)	207/4972 (4.2)	1.22 (0.68 to 2.21)	−0.03 (−0.14 to 0.09)
Lack of effective support staff	183/683 (26.8)	786/4984 (15.8)	1.70 (1.13 to 2.57)	−0.01 (−0.22 to 0.21)
Program not responsive to resident concerns	68/685 (9.9)	360/4965 (7.3)	1.21 (0.78 to 1.87)	0.03 (−0.11 to 0.16)
Program did not take wellness seriously	82/686 (12.0)	378/4978 (7.6)	1.53 (1.02 to 2.28)	0.01 (−0.13 to 0.15)

Abbreviations: IV, instrumental variable; OR, odds ratio.

^a^ Each row represents a separate model assessing the association of union status with each outcome. Residents with missing responses for the following outcomes were excluded from the model for that outcome: thoughts of attrition (n = 16), dissatisfaction with decision to become a surgeon (n = 12), dissatisfaction with time for rest (n = 9), duty hour violations (n = 118), discrimination (n = 414), bullying (n = 127), sexual harassment (n = 165), dissatisfaction with educational quality (n = 10), inadequate time for patient care (n = 33), lack of protected educational time (n = 64), inadequate time in operating room (n = 63), inadequate autonomy in operating room (n = 64), inadequate autonomy in clinical decisions (n = 47), lack of effective support staff (n = 34), program nonresponsiveness to resident concerns (n = 51), and program did not take wellness seriously (n = 37).

^b^ Logistic regression models estimated the OR for each outcome among residents at unionized programs with nonunionized programs as the reference group. Covariates included sex, race, Hispanic ethnicity, relationship status, parental status, census region, urban-rural classification, and program size. Odds ratios greater than 1.00 indicate worse outcome at unionized programs.

^c^ Instrumental variable regression models were estimated as linear probability models using regional rates of public sector employees who report being covered by labor unions as an IV for presence of a resident union. The same covariates used in logistic regression models were included. Coefficients represent the difference in probability of experiencing the outcome; values greater than 0 indicate worse outcomes at unionized programs.

^d^ Includes discrimination based on sex, gender identity, sexual orientation, race, ethnicity, and religion.

^e^ Remains significant after Bonferroni adjustment for multiple comparisons.

### Educational Environment

Residents at unionized programs responded more negatively to questions about the educational environment than nonunionized residents, more frequently reporting dissatisfaction with educational quality (105 of 688 [15.3%] vs 456 of 5003 [9.1%]; OR, 1.49 [95% CI, 1.03-2.17]), inadequate time for patient care (99 of 686 [14.4%] vs 458 of 4982 [9.2%]; OR, 1.38 [95% CI, 1.01-1.90]), a lack of effective support staff (183 of 683 [26.8%] vs 786 of 4984 [15.8%]; OR, 1.70 [95% CI, 1.13-2.57]), and the perception that their program did not take wellness seriously (82 of 686 [12.0%] vs 378 of 4978 [7.6%]; OR, 1.53 [95% CI, 1.02-2.28]) (Table 2). However, after addressing potential endogeneity with IV analysis, no significant differences remained. No differences were seen in a lack of protected educational time (124 of 678 [18.3%] vs 610 of 4959 [12.3%]), inadequate operating room time (93 of 681 [13.7%] vs 363 of 4957 [7.3%]), inadequate operative (83 of 682 [12.2%] vs 427 of 4955 [8.6%]) or clinical (37 of 682 [5.4%] vs 207 of 4972 [4.2%]) autonomy, or program nonresponsiveness to resident concerns (68 of 685 [9.9%] vs 360 of 4965 [7.3%]). These findings were consistent on IV analysis.

### Salary and Benefits

Salary did not significantly differ between unionized (mean [SD], $61 932 [$4557]) and nonunionized (mean [SD], $57 798 [$4652]) programs (Table 3). Unionized programs more frequently offered 4 weeks of vacation (27 of 29 [93.1%] vs 52 of 170 [30.6%]; OR, 19.18 [95% CI, 3.92-93.81]), which persisted on IV regression (difference in probability, 0.77 [95% CI, 0.09-1.45]), and housing stipends (10 of 26 [38.5%] vs 9 of 56 [16.1%]); this outcome was not significant on logistic regression (OR, 2.15 [95% CI, 0.58-7.95]) but was on IV analysis (difference in probability, 0.62 [95% CI, 0.04-1.20]). Conversely, rates of subsidized childcare (0 of 29 vs 16 of 170 [9.4%]), relocation stipends (2 of 29 [6.9%] vs 20 of 170 [11.8%]), and technology stipends (13 of 29 [44.8%] vs 76 of 170 [44.7%]) did not differ.

**Table 3.** Residency Program Outcomes for Unionized and Nonunionized Programs^a^

Outcome	Program, No./total No. (%)		Linear regression, OR or mean difference (95% CI)^b^^,^^c^	IV analysis, difference in probability (95% CI)^d^^,^^e^
	Unionized	Nonunionized		
PGY-1 salary, mean (SD), $	61 932 (4557)	57 798 (4652)	Mean difference, 552 (−1115 to 2220)	7180 (−2182 to 16 542)
Vacation length, wk				
<4	2/29 (6.9)	118/170 (69.4)	OR, 19.18 (3.92 to 93.81)^f^	0.77 (0.09 to 1.45)
4	27/29 (93.1)	52/170 (30.6)		
Subsidized childcare	0/29	16/170 (9.4)	NE	−0.07 (−0.50 to 0.37)
Housing stipend	10/26 (38.5)	9/56 (16.1)	OR, 2.15 (0.58 to 7.95)	0.62 (0.04 to 1.20)
Relocation stipend	2/29 (6.9)	20/170 (11.8)	OR, 1.13 (0.16 to 8.18)	0.24 (−0.26 to 0.74)
Technology stipend	13/29 (44.8)	76/170 (44.7)	OR, 0.85 (0.34 to 2.12)	−0.60 (−1.46 to 0.26)

Abbreviations: IV, instrumental variable; NE, not estimable; OR, odds ratio; PGY, postgraduate level.

^a^ Each row represents a separate model assessing the association of unionization status with each outcome. Nine programs without available salary data were excluded, resulting in 277 programs available for analysis. For benefits, only programs for which data were available through the American Medical Association FREIDA database or a publicly available union contract were included, resulting in 199 programs available for analysis (29 unionized and 170 nonunionized).

^b^ Linear regression model of salary for unionized vs nonunionized programs included program size, program type, census region, urban-rural classification, and county median household income as covariates.

^c^ Logistic regression models of unionized vs nonunionized programs included program size, program type, and urban-rural classification as covariates. For the housing stipend model, county-level median rent was also included.

^d^ Model estimated as a linear regression model salary for unionized vs nonunionized programs using regional rates of public sector employees covered by labor unions as an instrument for program unionization. The same covariates from the multivariable linear model were included.

^e^ Models estimated as linear probability models for unionized vs nonunionized programs using regional rates of public sector employees covered by labor unions as an instrument for program unionization. The same covariates used in logistic models were included. Coefficients represent the difference in probability of the outcome.

^f^ Remains significant after Bonferroni adjustment for multiple comparisons.

### Stratified Analysis

When stratified by location in the New York–Newark CSA (42 programs; 17 unionized and 25 nonunionized), findings similar to those of the primary analysis were seen, with certain exceptions (Table 4 and Table 5). Sexual harassment was reported less frequently at unionized programs within the New York–Newark CSA (70 of 326 [21.5%] vs 149 of 494 [30.2%]; OR, 0.64 [95% CI, 0.44-0.95]) but not outside that region (100 of 342 [29.2%] vs 1376 of 4374 [31.5%]). Within the New York–Newark CSA, residents at unionized programs more frequently reported inadequate operating room time (56 of 333 [16.8%] vs 44 of 493 [8.9%]; OR, 2.57 [95% CI, 1.16-5.69]) and a lack of effective support staff (108 of 333 [32.4%] vs 89 of 498 [17.9%]; OR, 2.94 [95% CI, 1.27-6.81]); however, these findings did not persist outside that region. Outside the New York–Newark CSA, residents at unionized programs more frequently reported dissatisfaction with educational quality (53 of 352 [15.1%] vs 393 of 4500 [8.7%]; OR, 1.74 [95% CI, 1.04-2.88]), inadequate time for patient care (58 of 351 [16.5%] vs 405 of 4484 [9.0%]; OR, 1.70 [95% CI, 1.09-2.65]), and the perception that their program did not take wellness seriously (46 of 351 [13.1%] vs 326 of 4480 [7.3%]; OR, 2.37 [95% CI, 1.43-3.95]). These associations were not seen within the New York–Newark CSA. Because the IV in this study is geographically based, IV methods could not be used for geographically stratified analyses

**Table 4.** Association Between Unionization Status and Resident Outcomes, Stratified by Location in the New York–Newark CSA^a^

Outcome	Within New York–Newark CSA			Outside New York–Newark CSA		
	Resident group, No./total No. (%)		Logistic regression, OR (95% CI)^b^	Resident group, No./total No. (%)		Logistic regression, OR (95% CI)^b^
	Unionized	Nonunionized		Unionized	Nonunionized	
Burnout	124/338 (36.7)	199/504 (39.5)	1.02 (0.73-1.42)	173/352 (49.1)	1976/4507 (43.8)	0.99 (0.74-1.34)
Suicidal ideation	11/338 (3.3)	18/504 (3.6)	0.72 (0.34-1.50)	15/352 (4.3)	216/4507 (4.8)	0.71 (0.37-1.33)
Job satisfaction						
Thoughts of attrition	39/338 (11.5)	53/504 (10.5)	1.22 (0.78-1.91)	50/351 (14.3)	525/4492 (11.7)	1.08 (0.73-1.60)
Dissatisfied with decision to become a surgeon	18/333 (5.4)	17/503 (3.4)	1.74 (0.85-3.54)	19/352 (5.4)	236/4501 (5.2)	0.90 (0.52-1.56)
Dissatisfied with time for rest	61/336 (18.2)	100/503 (19.9)	1.01 (0.61-1.69)	91/352 (25.9)	797/4501 (17.7)	1.35 (0.86-2.10)
Duty hour violations	124/324 (38.3)	218/486 (44.9)	0.80 (0.48-1.34)	175/348 (50.3)	1861/4425 (42.1)	1.07 (0.74-1.56)
Mistreatment						
Any discrimination^c^	156/309 (50.5)	245/464 (52.8)	0.85 (0.58-1.23)	188/321 (58.6)	2245/4193 (53.5)	0.99 (0.79-1.25)
Bullying	214/322 (66.5)	314/489 (64.2)	1.19 (0.81-1.76)	227/346 (65.6)	2980/4417 (67.5)	0.82 (0.63-1.07)
Sexual harassment	70/326 (21.5)	149/494 (30.2)	0.64 (0.44-0.95)	100/342 (29.2)	1376/4374 (31.5)	0.85 (0.63-1.16)
Educational environment						
Dissatisfied with educational quality	52/336 (15.5)	63/503 (12.5)	1.32 (0.66-2.64)	53/352 (15.1)	393/4500 (8.7)	1.74 (1.04-2.88)
Inadequate time for patient care	41/335 (12.2)	53/498 (10.6)	1.28 (0.71-2.31)	58/351 (16.5)	405/4484 (9.0)	1.70 (1.09-2.65)
Lack of protected educational time	51/328 (15.6)	71/496 (14.3)	1.27 (0.60-2.65)	73/350 (20.9)	539/4463 (12.1)	1.78 (0.93-3.41)
Inadequate time in operating room	56/333 (16.8)	44/493 (8.9)	2.57 (1.16-5.69)	37/348 (10.6)	319/4464 (7.2)	1.14 (0.60-2.16)
Inadequate autonomy in operating room	41/331 (12.4)	62/494 (12.6)	1.14 (0.51-2.55)	42/351 (12.0)	365/4461 (8.2)	1.28 (0.69-2.38)
Inadequate autonomy in clinical decisions	21/331 (6.3)	29/497 (5.8)	1.05 (0.44-2.51)	16/351 (4.6)	178/4475 (4.0)	1.30 (0.56-2.99)
Lack of effective support staff	108/333 (32.4)	89/498 (17.9)	2.94 (1.27-6.81)	75/350 (21.4)	697/4486 (15.5)	1.36 (0.72-2.58)
Program not responsive to resident concerns	35/335 (10.5)	49/498 (9.8)	1.22 (0.63-2.35)	33/350 (9.4)	311/4467 (7.0)	1.49 (0.80-2.79)
Program did not take wellness seriously	36/335 (10.8)	52/498 (10.4)	1.15 (0.61-2.17)	46/351 (13.1)	326/4480 (7.3)	2.37 (1.43-3.95)^d^

Abbreviations: CSA, combined statistical area; OR, odds ratio.

^a^ Because 56.7% of unionized programs are located in the New York–Newark CSA, this sensitivity analysis assesses unionization stratified by program location within that area. Separate models were used to estimate each outcome among programs inside and outside the New York–Newark CSA. Residents with missing responses for the following outcomes were excluded from the model for that outcome: thoughts of attrition (n = 16), dissatisfaction with decision to become a surgeon (n = 12), dissatisfaction with time for rest (n = 9), duty hour violations (n = 118), discrimination (n = 414), bullying (n = 127), sexual harassment (n = 165), dissatisfaction with educational quality (n = 10), inadequate time for patient care (n = 33), lack of protected educational time (n = 64), inadequate time in operating room (n = 63), inadequate autonomy in operating room (n = 64), inadequate autonomy in clinical decisions (n = 47), lack of effective support staff (n = 34), program nonresponsiveness to resident concerns (n = 51), and program did not take wellness seriously (n = 37).

^b^ Logistic regression models estimating ORs for residents at unionized vs nonunionized programs. Covariates included sex, race, Hispanic ethnicity, and program size. For the models of programs outside the New York–Newark CSA, census region and urban-rural classification were also included. Odds ratios greater than 1.00 indicate worse outcome for unionized programs.

^c^ Includes discrimination based on sex, gender identity, sexual orientation, race, ethnicity, and religion.

^d^ Remains significant after Bonferroni correction for multiple comparisons.

**Table 5.** Association Between Unionization Status and Program Outcomes, Stratified by Location in the New York–Newark CSA^a^

Outcome	Programs in New York–Newark CSA			Programs outside New York–Newark CSA		
	Program type^b^		Linear regression, mean difference or OR (95% CI)^c^^,^^d^	Program type^b^		Linear regression, mean difference or OR (95% CI)^c^^,^^d^
	Unionized	Nonunionized		Unionized	Nonunionized	
Salary, mean (SD), US$^e^	63 369 (3059)	65 525 (6437)	Mean difference, −2723 (−6145 to 700)	60 164 (5532)	57 045 (3671)	Mean difference, 213 (−1895 to 2322)
Vacation length, wk						
No.	17	15		12	155	
<4	0/17	4/15 (26.7)	NE	2/12 (16.7)	114/155 (73.5)	OR, 11.05 (1.83 to 66.70)
4	17/17 (100)	11/15 (73/3)		10/12 (83.3)	41/155 (26.5)	
Subsidized childcare	0/17	1/15 (6.7)	NE	0/12	15/155 (9.7)	NE
Housing stipend	4/17 (23.5)	8/15 (53.3)	OR, 0.24 (0.05 to 1.20)	6/12 (50.0)	2/155 (1.3)	OR, 1.71 (0.11 to 25.98)
Relocation stipend	0/17	0/15	NE	2/12 (16.7)	20/155 (12.9)	OR, 2.75 (0.27 to 28.33)
Technology stipend	9/17 (52.9)	10/15 (66.7)	OR, 0.57 (0.13 to 2.45)	4/12 (33.3)	66/155 (42.6)	OR, 0.54 (0.12 to 2.46)

Abbreviations: CSA, combined statistical area; NE, not estimable; OR, odds ratio.

^a^ Because 56.7% of unionized programs are within the New York–Newark CSA, this sensitivity analysis assesses unionization stratified by program location within vs outside that area. Separate models were used to estimate each outcome among programs inside and outside the New York–Newark CSA. Nine programs without available salary data were excluded, resulting in 277 programs available for analysis. For benefits, only programs for which data were available through the American Medical Association FREIDA database or a publicly available union contract were included, resulting in 199 programs available for analysis.

^b^ Unless otherwise indicated, data are expressed as number/total number (percentage) of programs.

^c^ Linear regression model of salary for unionized vs nonunionized programs including program size, program type, and county median household income as covariates. For programs outside the New York–Newark CSA, geographic region and urban-rural classification were also included as covariates.

^d^ Logistic regression models of unionized vs nonunionized programs including program size and program type as covariates. For programs outside the New York–Newark CSA, geographic region and urban-rural classification were also included as covariates. For the housing stipend model, county-level median rent was also included.

^e^ Includes 16 unionized and 22 nonunionized programs within the New York–Newark CSA and 13 unionized and 226 nonunionized programs outside the New York–Newark CSA.

### Sensitivity Analyses

With burnout defined as a continuous variable, no difference in burnout score was seen between unionized programs (mean [SD], 10.6 [8.6]) and nonunionized programs (mean [SD], 10.8 [8.1]; naive adjusted mean difference, −0.4 [95% CI, −1.3 to 0.5]; IV adjusted mean difference, 2.1 [95% CI, −2.8 to 6.9]). In addition, when comparing programs unionized for more than 3 years against those that were not, similar findings to the primary analyses were observed (eTables 3 and 4 in the Supplement). Estimates derived from naive linear probability models were similar to those obtained from logistic regression models (eTable 5 in the Supplement).

## Discussion

This study was undertaken to assess the association of resident unions with well-being, educational environment, salary, and benefits among surgical residents. In this US study of 5701 surgical residents, unionization was not associated with improvements in burnout, suicidality, job satisfaction, duty hour violations, mistreatment, educational environment, or salary but was associated with better vacation and housing stipend benefits. These results should be considered as residents and residency programs contemplate unionization.

### Burnout, Suicidality, Job Satisfaction, Duty Hour Violations, and Mistreatment

Despite claims that unions improve resident working conditions and well-being,^14,16^ we found no association between unionized programs and improvements in burnout, suicidality, job satisfaction, or duty hour violations. Sexual harassment was reported less frequently among unionized residents, but this finding did not persist on IV analysis. This result raises the possibility that reduced rates of sexual harassment may not be attributable to unions but rather may be an artifact of unmeasured confounders, such as a culture that discourages sexual harassment and supports unionization.

### Educational Environment

Multiple educational environment measures were inferior at unionized programs on naive analysis, including dissatisfaction with educational quality, inadequate time for patient care, lack of effective support staff, and a perception that the program did not take wellness seriously. However, these associations were no longer apparent on IV analysis. Instrumental variable analysis is essential to evaluate the causal association between unionization and the study outcomes because naive models are susceptible to unmeasured confounders (eg, cultural factors that may accompany support for unionization) as well as reverse causality, in which a poor educational environment may prompt residents to pursue unionization.

### Salary and Benefits

Unionized programs were significantly more likely to offer 4 weeks of vacation. This finding remained on IV analysis, suggesting that unions indeed secure increased vacation time. In addition, after controlling for local housing costs, housing stipends were more common among unionized programs on IV analysis. These findings are unsurprising given that contract negotiation is a central function of labor unions. Nonetheless, despite these tangible benefits, no differences were seen in well-being measures between unionized and nonunionized programs. This outcome may be attributable in part to union membership dues, which may offset some of the financial benefits afforded by unionization.

Several potential reasons explain why few outcomes were associated with unionization in this study. First, burnout is a complex entity resulting from multiple factors, and the effect of unions on a small number of these factors may be insufficient to meaningfully reduce burnout. However, the fact that no outcomes aside from vacation duration and housing stipends differed among unionized programs suggests that the multifaceted nature of burnout alone is not likely to explain the lack of an observed benefit. Second, resident unions have limited leverage compared with other labor unions. Because of patient safety concerns and residents’ unique positions as trainees who depend on their leadership for career advancement, strikes are rarely used, leaving fewer mechanisms to induce change. In addition, resident compensation is linked to standardized payments from Medicare and other entities, potentially limiting the ability of unions to negotiate salaries. Third, unions are typically institution wide and may focus on issues that are not specifically relevant to surgical trainees, particularly if surgical specialties are underrepresented in union leadership. Fourth, the introduction of duty hour regulations and increased awareness of workplace mistreatment may have improved conditions that otherwise would have been addressed by labor unions. Residents at unionized programs were previously shown to receive improved wages and benefits, but this advantage deteriorated from 1979 to 1983; the benefit of unions may have declined further since.^37^ Fifth, the adverse effects of unionization may counteract potential benefits. Union members generally must pay annual dues, which may partially offset financial benefits. In addition, union formation and periodic contract negotiations may lead to an adversarial relationship between leadership and residents, with increasing attention drawn to perceived institutional shortcomings, whether or not they differ from those of a nonunionized institution.^18^ Last, it is possible that unions simply do not improve the measured outcomes.

### Limitations

This study has several limitations. First, this is a cross-sectional rather than a longitudinal survey study. Although IV techniques approximate causality, causal associations between unionization and the study outcomes cannot be definitively ascertained. Second, unionized programs are heavily concentrated in major metropolitan areas. To address potential confounding, geographic region and urban-rural classification were included in models, IV analysis was used, and analyses were stratified by location in the New York–Newark CSA. Although stratified analyses suggest that the association of unions with several outcomes may vary by location, causality cannot be presumed without IV techniques. Third, the use of a geographically based instrument results in decreased statistical power. Although a program-level instrument (eg, unionization of other employees at the primary training site) would be preferable, such an instrument would likely be related to workplace culture and resident outcomes independently of the presence of a resident union and therefore would not satisfy the conditions of a valid instrument. For this reason, geographically based IVs are widely used in the literature.^38,39,40,41^ Fourth, burnout prevalence estimates derived from the burnout definition used in this study cannot be compared with different definitions used in other studies. Furthermore, the aMBI measure used in this study is slightly less robust than the full-length MBI, although this factor is not expected to substantially alter the findings of this study. Fifth, outcomes such as institutional culture and resident-institution cooperation could not be assessed in this study. Sixth, because IV models are less precise than naive regression models, they may not be as sensitive in identifying subtle differences. Seventh, residents were surveyed immediately after the ABSITE, and their responses may be affected by the stress of this setting. Last, it is unknown whether the findings of this study are generalizable to other fields. Because surgical residents are at a particularly high risk for burnout, mistreatment, and duty hour violations,^27,42,43,44^ any benefits of unionization would likely be amplified in this high-risk specialty, suggesting that the findings of this study may be applicable beyond surgical trainees.

## Conclusions

To our knowledge, this is the first US study to evaluate the association of resident unions with working conditions and well-being. This study shows that vacation time and housing stipend benefits are improved at unionized programs; however, unions do not appear to improve burnout, suicidality, job satisfaction, duty hour violations, mistreatment, or program educational environment. These findings should be noted as residents and residency programs discuss resident well-being and contemplate unionization.

## Notes

Supplement. **eFigure.** Response Rate Calculation **eMethods 1.** Selected Items From Survey Administered to Residents After the 2019 ABSITE **eMethods 2.** Rationale for Development of Instrumental Variable Regression Model **eTable 1.** Supplementary Statistics From First Stage of Instrumental Variable Regression Models **eTable 2.** Characteristics of Survey Respondents and Nonrespondents **eTable 3.** Resident Outcomes for Trainees at Programs That Have Been Unionized for 3 or More Years **eTable 4.** Residency Program Outcomes for Programs That Have Been Unionized for 3 or More Years **eTable 5.** Comparison of the Association Between Unionization Status and Outcome Using Linear Probability Models

## References

[1] Shanafelt TD, Boone S, Tan L, . Burnout and satisfaction with work-life balance among US physicians relative to the general US population. Arch Intern Med. 2012;172(18):1377-1385. doi:10.1001/archinternmed.2012.3199 22911330

[2] Hu YY, Ellis RJ, Hewitt DB, . Discrimination, abuse, harassment, and burnout in surgical residency training. N Engl J Med. 2019;381(18):1741-1752. doi:10.1056/NEJMsa1903759 31657887 PMC6907686

[3] World Health Organization. International Classification of Diseases, 11th Revision. World Health Organization; 2019. Accessed January 20, 2021. https://icd.who.int/en

[4] Shanafelt TD, Balch CM, Bechamps G, . Burnout and medical errors among American surgeons. Ann Surg. 2010;251(6):995-1000. doi:10.1097/SLA.0b013e3181bfdab3 19934755

[5] West CP, Shanafelt TD, Kolars JC. Quality of life, burnout, educational debt, and medical knowledge among internal medicine residents. JAMA. 2011;306(9):952-960. doi:10.1001/jama.2011.1247 21900135

[6] Lebares CC, Guvva EV, Ascher NL, O’Sullivan PS, Harris HW, Epel ES. Burnout and stress among US surgery residents: psychological distress and resilience. J Am Coll Surg. 2018;226(1):80-90. doi:10.1016/j.jamcollsurg.2017.10.010 29107117

[7] Elmore LC, Jeffe DB, Jin L, Awad MM, Turnbull IR. National survey of burnout among US general surgery residents. J Am Coll Surg. 2016;223(3):440-451. doi:10.1016/j.jamcollsurg.2016.05.014 27238875 PMC5476455

[8] Shanafelt TD, Hasan O, Dyrbye LN, . Changes in burnout and satisfaction with work-life balance in physicians and the general US working population between 2011 and 2014. Mayo Clin Proc. 2015;90(12):1600-1613. doi:10.1016/j.mayocp.2015.08.023 26653297

[9] Dyrbye LN, Massie FS Jr, Eacker A, . Relationship between burnout and professional conduct and attitudes among US medical students. JAMA. 2010;304(11):1173-1180. doi:10.1001/jama.2010.1318 20841530

[10] McManus IC, Winder BC, Gordon D. The causal links between stress and burnout in a longitudinal study of UK doctors. Lancet. 2002;359(9323):2089-2090. doi:10.1016/S0140-6736(02)08915-8 12086767

[11] Shanafelt TD, Dyrbye LN, West CP. Addressing physician burnout: the way forward. JAMA. 2017;317(9):901-902. doi:10.1001/jama.2017.0076 28196201

[12] Bennett JT, Kaufman BE. What do unions do? a twenty-year perspective. J Labor Res. 2004;25(3):339-349. doi:10.1007/s12122-004-1020-y

[13] Hagedorn J, Paras CA, Greenwich H, Hagopian A. The role of labor unions in creating working conditions that promote public health. Am J Public Health. 2016;106(6):989-995. doi:10.2105/AJPH.2016.303138 27077343 PMC4880255

[14] Committee of Interns and Residents. Committee of Interns and Residents. 2019. Accessed September 1, 2020. https://www.cirseiu.org/

[15] Smith MJ. Should surgical residents unionize? *General Surgery News*. December 15, 2020. Accessed December 23, 2020. https://www.generalsurgerynews.com/In-the-News/Article/12-20/Should-Surgical-Residents-Unionize-/61397

[16] Krupp L. Resident physician abuse: are unions the answer? *Medscape*. August 4, 2020. Accessed January 21, 2021. https://www.medscape.com/viewarticle/935149

[17] Yacht AC. Collective bargaining is the right step. N Engl J Med. 2000;342(6):429-431. doi:10.1056/NEJM200002103420613 10666437

[18] Cohen JJ. White coats should not have union labels. N Engl J Med. 2000;342(6):431-434. doi:10.1056/NEJM200002103420614 10666438

[19] American Association for Public Opinion Research (AAPOR). Standard definitions: final dispositions of case codes and outcome rates for surveys, 9th Edition. Revised 2016. Accessed February 2, 2021. http://www.aapor.org/AAPOR_Main/media/publications/Standard-Definitions20169theditionfinal.pdf

[20] Zhang LM, Cheung EO, Eng JS, . Development of a conceptual model for understanding the learning environment and surgical resident well-being. Am J Surg. 2021;221(2):323-330. doi:10.1016/j.amjsurg.2020.10.026 33121657

[21] Shanafelt TD, Balch CM, Dyrbye L, . Special report: suicidal ideation among American surgeons. Arch Surg. 2011;146(1):54-62. doi:10.1001/archsurg.2010.292 21242446

[22] Bilimoria KY, Chung JW, Hedges LV, . National cluster-randomized trial of duty-hour flexibility in surgical training. N Engl J Med. 2016;374(8):713-727. doi:10.1056/NEJMoa1515724 26836220

[23] Bilimoria KY, Chung JW, Hedges LV, . Development of the Flexibility in Duty Hour Requirements for Surgical Trainees (FIRST) trial protocol: a national cluster-randomized trial of resident duty hour policies. JAMA Surg. 2016;151(3):273-281. doi:10.1001/jamasurg.2015.4990 26720622

[24] Desai SV, Asch DA, Bellini LM, ; iCOMPARE Research Group. Education outcomes in a duty-hour flexibility trial in internal medicine. N Engl J Med. 2018;378(16):1494-1508. doi:10.1056/NEJMoa1800965 29557719 PMC6101652

[25] Maslach C, Jackson S, Leiter M, Schaufeli W, Schwab R. Maslach Burnout Inventory Manual. *4th ed*. Mind Garden Inc; 2016.

[26] National Center for Health Statistics. NCHS Urban-Rural Classification Scheme for Counties. Reviewed June 1, 2017. Accessed February 1, 2021. https://www.cdc.gov/nchs/data_access/urban_rural.htm

[27] Riley MR, Mohr DC, Waddimba AC. The reliability and validity of three-item screening measures for burnout: evidence from group-employed health care practitioners in upstate New York. Stress Health. 2018;34(1):187-193. doi:10.1002/smi.2762 28524379

[28] Dyrbye LN, Burke SE, Hardeman RR, . Association of clinical specialty with symptoms of burnout and career choice regret among US resident physicians. JAMA. 2018;320(11):1114-1130. Retracted in: *JAMA*. 2019;321(12):1220-1221. doi:10.1001/jama.2018.12615 30912842

[29] Hewitt DB, Ellis RJ, Hu YY, . Evaluating the association of multiple burnout definitions and thresholds with prevalence and outcomes. JAMA Surg. 2020;155(11):1043-1049. doi:10.1001/jamasurg.2020.3351 32902609 PMC7489413

[30] Schaufeli WB, Bakker AB, Hoogduin K, Schaap C, Kladler A. On the clinical validity of the Maslach Burnout Inventory and the burnout measure. Psychol Health. 2001;16(5):565-582. doi:10.1080/08870440108405527 22804499

[31] American Medical Association. FREIDA. Published 2021. Accessed November 18, 2020. https://freida.ama-assn.org/

[32] US Bureau of Labor Statistics. Databases, tables, & calculators by subject. Updated December 2020. Accessed December 7, 2020. https://www.bls.gov/data/

[33] US Census Bureau. American Community Survey (ACS). 2020. Accessed December 7, 2020. https://www.census.gov/programs-surveys/acs

[34] Angrist JD, Krueger AB. Instrumental variables and the search for identification: from supply and demand to natural experiments. J Econ Perspect*.* 2001;15(4):69-85. doi:10.1257/jep.15.4.69

[35] Hirsch BT, MacPherson DA. Union membership and coverage database from the current population survey: note. Ind Labor Relat Rev*.* 2003;56(2):349-354. doi:10.1177/001979390305600208

[36] US Census Bureau. Design and methodology: current population survey—America's source for labor force data, Technical Paper 77. October 2019. Accessed December 1, 2020. https://www2.census.gov/programs-surveys/cps/methodology/CPS-Tech-Paper-77.pdf

[37] Bazzoli GJ. Changes in resident physicians’ collective bargaining outcomes as union strength declines. Med Care. 1988;26(3):263-277. doi:10.1097/00005650-198803000-00004 3352324

[38] McClellan M, McNeil BJ, Newhouse JP. Does more intensive treatment of acute myocardial infarction in the elderly reduce mortality? analysis using instrumental variables. JAMA. 1994;272(11):859-866. doi:10.1001/jama.1994.03520110039026 8078163

[39] Newhouse JP, McClellan M. Econometrics in outcomes research: the use of instrumental variables. Annu Rev Public Health. 1998;19:17-34. doi:10.1146/annurev.publhealth.19.1.17 9611610

[40] Stukel TA, Fisher ES, Wennberg DE, Alter DA, Gottlieb DJ, Vermeulen MJ. Analysis of observational studies in the presence of treatment selection bias: effects of invasive cardiac management on AMI survival using propensity score and instrumental variable methods. JAMA. 2007;297(3):278-285. doi:10.1001/jama.297.3.278 17227979 PMC2170524

[41] Landrum MB, Ayanian JZ. Causal effect of ambulatory specialty care on mortality following myocardial infarction: a comparison of propensity score and instrumental variable analyses. Health Serv Outcomes Res Methodo*l*. 2001;2(3):221-245. doi:10.1023/A:1020367111374

[42] Frank E, Brogan D, Schiffman M. Prevalence and correlates of harassment among US women physicians. Arch Intern Med. 1998;158(4):352-358. doi:10.1001/archinte.158.4.352 9487232

[43] Fnais N, Soobiah C, Chen MH, . Harassment and discrimination in medical training: a systematic review and meta-analysis. Acad Med. 2014;89(5):817-827. doi:10.1097/ACM.0000000000000200 24667512

[44] National Academies of Sciences, Engineering, and Medicine. Sexual Harassment of Women: Climate, Culture, and Consequences in Academic Sciences, Engineering, and Medicine. National Academies Press; 2018. 29894119

