# Supplementary material for: National Evaluation of the Association Between Resident Labor Union Participation and Surgical Resident Well-being
Source: JAMA Netw Open. 2021 Sep 1;4(9):e2123412. doi: 10.1001/jamanetworkopen.2021.23412 (PMC8411294; doi:10.1001/jamanetworkopen.2021.23412)
Supplement: Supplement. — eFigure. Response Rate Calculation eMethods 1. Selected Items From Survey Administered to Residents After the 2019 ABSITE eMethods 2. Rationale for Development of Instrumental Variable Regression Model eTable 1. Supplementary Statistics From First Stage of Instrumental Variable Regression Models eTable 2. Characteristics of Survey Respondents and Nonrespondents eTable 3. Resident Outcomes for Trainees at Programs That Have Been Unionized for 3 or More Years eTable 4. Residency Program Outcomes for Programs That Have Been Unionized for 3 or More Years eTable 5. Comparison of the Association Between Unionization Status and Outcome Using Linear Probability Models [file jamanetwopen-e2123412-s001.pdf]

Supplementary Online Content

Brajcich BC, Chung JW, Wood DE, et al. National evaluation of the association between resident labor union participation and surgical resident well-being. *JAMA Netw Open*. 2021;4(9):e2123412. doi:10.1001/jamanetworkopen.2021.23412

eFigure. Response Rate Calculation

eMethods 1. Selected Items From Survey Administered to Residents After the 2019 ABSITE

eMethods 2. Rationale for Development of Instrumental Variable Regression Model

eTable 1. Supplementary Statistics From First Stage of Instrumental Variable Regression Models

eTable 2. Characteristics of Survey Respondents and Nonrespondents

eTable 3. Resident Outcomes for Trainees at Programs That Have Been Unionized for 3 or More Years

eTable 4. Residency Program Outcomes for Programs That Have Been Unionized for 3 or More Years

eTable 5. Comparison of the Association Between Unionization Status and Outcome Using Linear Probability Models

This supplementary material has been provided by the authors to give readers additional information about their work.

© 2021 Brajcich BC et al. *JAMA Network Open*.

eFigure. Response Rate Calculation

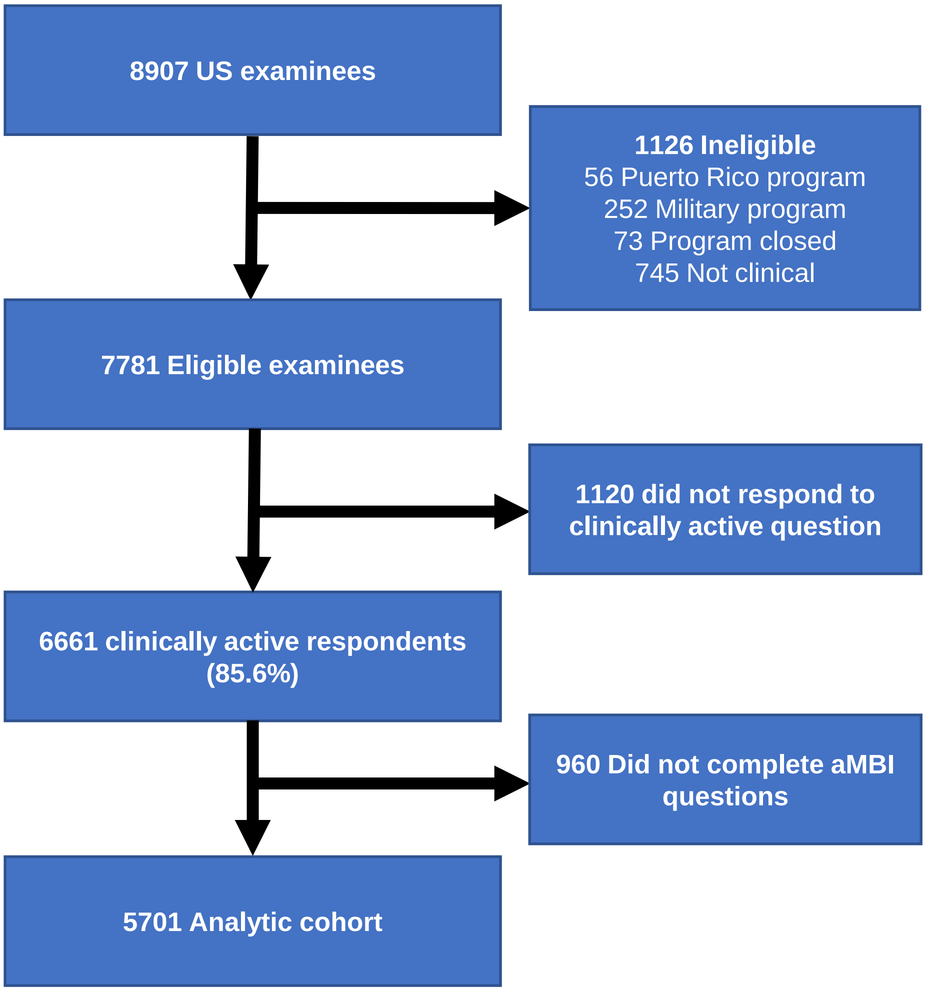

The survey response rate was calculated as the percentage of the 7781 eligible examinees who responded to the survey. Residents from Puerto Rican programs, military programs, programs which closed before unionization data was obtained, and non-clinically active residents were ineligible and were excluded from response rate calculations.

© 2021 Brajcich BC et al. *JAMA Network Open*.

eMethods 1. Selected Items From Survey Administered to Residents After the 2019 ABSITE

INSTRUCTIONS

Please complete the following survey to share your thoughts about your residency experience. The information will be used to inform future research and policy decisions to improve the learning environment and culture of residency. It is estimated that it will take approximately 5 to 8 minutes to complete the questions. **The survey responses are never associated with your personal identity. All data are de-identified for analyses and reporting. Your program will not have access to your individual responses.**

**1. Are you currently enrolled in a clinically active year of your residency? This is defined as the PGY1-5 years that count toward your board eligibility.**
☐ Yes
☐ No, I am currently working on research (full time), obtaining an additional degree (full time), or otherwise taking a break from my clinical years of training.

**2. The following describes me: (most applicable)**	
a. Married	☐
b. Not married but in a relationship	☐
c. Not married and not in a relationship (single)	☐
d. Divorced/Separated	☐
e. Widowed	☐

**3. As of July 2018, how many children (under 18) do you have?**	
a. 0	☐
b. 1	☐
c. 2	☐
d. 3	☐
e. 4	☐
f. 5+	☐

**4. During this academic year (July 2018 to present), were you or your partner pregnant/adopting/expecting a child?**	
Yes	No
☐	☐

**5. I identify my race as: (select all that apply)**	
a. American Indian or Alaska Native	☐
b. Asian	☐
c. Black or African American	☐
d. Native Hawaiian or Other Pacific Islander	☐
e. White	☐
f. Other	☐
g. Prefer not to say	☐

© 2021 Brajcich BC et al. *JAMA Network Open*.

**6. I identify my ethnicity as:**	
a. Hispanic or Latino	☐
b. Not Hispanic or Latino	☐
c. Prefer not to say	☐

7. Overall, how many months have you violated the following duty hour regulations during this academic year (July 2018 to present)?

Item	0	1	2	3	4	5+
a. Maximum 80 hours of work per week, averaged over a four-week period (including all in-house clinical and educational activities, clinical work done from home, and all moonlighting).	☐	☐	☐	☐	☐	☐
b. At least 1 day off in 7, averaged over a four-week period.	☐	☐	☐	☐	☐	☐
c. In-house call no more frequently than 1 in 3 nights, averaged over a four-week period.	☐	☐	☐	☐	☐	☐

8. Please indicate the extent to which you agree with the following statements:

Item	Strongly disagree	Disagree	Neutral	Agree	Strongly agree
a. I have enough time for direct patient care after completion of administrative tasks (e.g., notes, orders).	☐	☐	☐	☐	☐
b. My program effectively uses support staff (e.g., advance practice providers, social workers, patient transporters, etc.) to allow me to spend more time on patient care activities.	☐	☐	☐	☐	☐
c. My program protects educational time.	☐	☐	☐	☐	☐
d. Relative to my training level, I spend an appropriate amount of time in the operating room.	☐	☐	☐	☐	☐
e. Relative to my training level, I have an appropriate level of operative autonomy.	☐	☐	☐	☐	☐
f. Relative to my training level, I have an appropriate level of autonomy in patient care and clinical decision-making.	☐	☐	☐	☐	☐
g. My program is responsive to resident concerns.	☐	☐	☐	☐	☐
h. Overall, I believe my program takes my wellness seriously.	☐	☐	☐	☐	☐

9. Thinking back on this academic year (July 2018 to present), how satisfied were you with the following?

Item	Very Dissatisfied	Dissatisfied	Neutral	Satisfied	Very Satisfied
a. Quality of overall resident education.	☐	☐	☐	☐	☐
b. Time for rest.	☐	☐	☐	☐	☐
c. Your decision to become a surgeon.	☐	☐	☐	☐	☐

© 2021 Brajcich BC et al. *JAMA Network Open*.

10. Using the scale below, indicate the frequency with which you've experienced the following in your general surgery residency during this academic year (July 2018 to present).					
**Item**	**Never**	**Now and then**	**Monthly**	**Weekly**	**Daily**
a. Someone withholding information which affects your performance	☐	☐	☐	☐	☐
b. Spreading of gossip and rumors about you	☐	☐	☐	☐	☐
c. Being ignored or excluded	☐	☐	☐	☐	☐
d. Having insulting or offensive remarks made about your person, attitudes, or your private life	☐	☐	☐	☐	☐
e. Being shouted at or being the target of spontaneous anger	☐	☐	☐	☐	☐
f. Repeated reminders of your errors or mistakes	☐	☐	☐	☐	☐
g. Being ignored or facing a hostile reaction when you approach	☐	☐	☐	☐	☐
h. Persistent criticism of your work and effort	☐	☐	☐	☐	☐
i. Practical jokes carried about by people you don't get along with	☐	☐	☐	☐	☐
j. Being cursed or sworn at	☐	☐	☐	☐	☐

11. Please indicate the extent to which you agree with the following statement:					
**Item**	**Strongly disagree**	**Disagree**	**Neutral**	**Agree**	**Strongly agree**
I have considered leaving my residency program during the current academic year (July 2018 to present).	☐	☐	☐	☐	☐

12. During the past <u>12 months</u>:		
	No	Yes
Have you had thoughts of taking your own life?	☐	☐

16. Using the scale below, indicate the frequency with which you've experienced the following in your general surgery residency during this academic year (July 2018 to present).							
**Item**	**Never**	**A few times a year**	**Once a month or less**	**A few times a month**	**Once a week**	**A few times a week**	**Every Day**
a. Crude/sexually demeaning or explicit remarks, stories, or jokes	☐	☐	☐	☐	☐	☐	☐
b. Unwanted sexual imagery or materials sent or shown to you	☐	☐	☐	☐	☐	☐	☐
c. Unwanted verbal sexual attention (e.g., comments, flirtations, sexual advances)	☐	☐	☐	☐	☐	☐	☐

d. Offensive body language (e.g., leering, standing too close)	☐	☐	☐	☐	☐	☐	☐
e. Unwanted physical sexual attention (e.g., inappropriate or uncomfortable touching; attempts to touch, fondle or kiss)	☐	☐	☐	☐	☐	☐	☐
f. Sexual coercion (e.g., bribed or threatened to engage in sexual behavior; suggestion of better treatment if sexually cooperative)	☐	☐	☐	☐	☐	☐	☐

17. Using the scale below, indicate the frequency with which you've experienced the following based on your <u>GENDER/GENDER IDENTITY/SEXUAL ORIENTATION</u> in your general surgery residency during this academic year (July 2018 to present). These questions are NOT related to race/ethnicity/religion.

Item	Never	A few times a year	Once a month or less	A few times a month	Once a week	A few times a week	Every Day
a. Different standards of evaluation (e.g., lowered expectations, need to work harder to achieve the same success as others, unfair punishment, less respect for my opinion)	☐	☐	☐	☐	☐	☐	☐
b. Denied opportunities (e.g., OR cases, attendance at conferences, career options, opportunities for advancements, mentorship)	☐	☐	☐	☐	☐	☐	☐
c. Mistaken for a non-physician based on your <u>gender/gender identity/sexual orientation</u>	☐	☐	☐	☐	☐	☐	☐
d. Slurs and/or hurtful/ humiliating/negative/uncomfortable comments even when purported as jokes	☐	☐	☐	☐	☐	☐	☐
e. Socially isolated (e.g., excluded from social events, malicious gossip)	☐	☐	☐	☐	☐	☐	☐
f. Was advised against having children during residency	☐	☐	☐	☐	☐	☐	☐
g. Felt like my/my partner's pregnancy or our childcare needs led to negative reactions from my coworkers/program	☐	☐	☐	☐	☐	☐	☐

© 2021 Brajcich BC et al. *JAMA Network Open*.

**18. Using the scale below, indicate the frequency with which you've experienced the following based on your <u>RACE/ETHNICITY/RELIGION</u> in your general surgery residency during this academic year (July 2018 to present). These questions are NOT related to gender/gender identity/sexual orientation.**							
**Item**	**Never**	**A few times a year**	**Once a month or less**	**A few times a month**	**Once a week**	**A few times a week**	**Every Day**
a. Different standards of evaluation (e.g., lowered expectations, need to work harder to achieve the same success as others, unfair punishment, less respect for my opinion)	☐	☐	☐	☐	☐	☐	☐
b. Denied opportunities (e.g., OR cases, attendance at conferences, career options, opportunities for advancements, mentorship)	☐	☐	☐	☐	☐	☐	☐
c. Mistaken for a non-physician based on your <u>race/ethnicity/religion</u>	☐	☐	☐	☐	☐	☐	☐
d. Slurs and/or hurtful/humiliating/negative/uncomfortable comments, even when purported as jokes	☐	☐	☐	☐	☐	☐	☐
e. Socially isolated (e.g., excluded from social events, malicious gossip)	☐	☐	☐	☐	☐	☐	☐
f. Mistaken for another person of the same race	☐	☐	☐	☐	☐	☐	☐

© 2021 Brajcich BC et al. *JAMA Network Open*.

eMethods 2: Rationale for Development of Instrumental Variable Regression Model

An instrumental variable modeling approach was employed in this study to account for suspected endogeneity of the variable denoting whether a program was unionized. Sources of endogeneity were hypothesized to include unmeasured confounding program characteristics which may influence the decision to unionize, and simultaneity, where outcomes related to poor working conditions or well-being may have contributed to the decision to form a union. The unionization rate of non-healthcare employees within a given region was selected as an instrumental variable for this study because it meets the following criteria:

1. It is strongly associated with the unionization status of residency programs within the region, irrespective of other confounding variables.

2. It is not itself associated with the outcomes of interest though any mechanism except program unionization status, as it does not directly affect outcomes (the instrument does not measure healthcare worker unions) and other regional confounding factors (geographic region, urban-rural classification, and where pertinent, income and housing costs) are included in the models.

The causal graph of the model is depicted below:

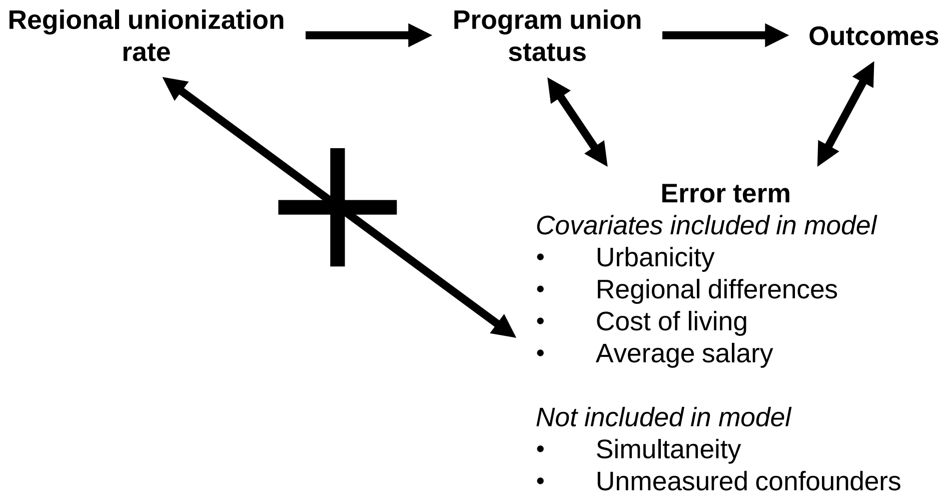

Included within the error term in the causal graph are several measured covariates which are incorporated as adjustments in models. After adjusting for these covariates, no association between the instrument (regional unionization rate) and the outcomes is believed to be present except through the primary exposure variable (program union status). The instrumental variable models used in this study can theoretically identify the direct influence of program unionization status on the outcomes independent from other unmeasured confounders (such as individual program characteristics that may confound union status and outcomes) and simultaneity, which the naïve regression models included in the manuscript are unable to do.

© 2021 Brajcich BC et al. *JAMA Network Open*.

eTable 1. Supplementary Statistics From First Stage of Instrumental Variable Regression Models

Outcome	First Stage F Statistic[a]
Burnout	14.30
Suicidal ideation	14.30
Job satisfaction	
Thoughts of attrition	14.26
Dissatisfied with decision to become a surgeon	14.18
Dissatisfied with time for rest	14.26
Duty hour violations	14.37
Mistreatment	
Any discrimination	14.18
Bullying	14.07
Sexual harassment	14.44
Educational environment	
Dissatisfied with educational quality	14.27
Inadequate time for patient care	14.35
Lack of protected educational time	14.31
Inadequate time in operating room	14.44
Inadequate autonomy in operating room	14.23
Inadequate autonomy in clinical decisions	14.22
Lack of effective support staff	14.28
Program not responsive to resident concerns	14.32
Program did not take wellness seriously	14.28
PGY-1 salary	9.04
Vacation length	14.74
Subsidized childcare	14.74
Housing stipend	11.31
Relocation stipend	14.74
Technology stipend	14.74

Abbreviations: PGY, post-graduate year

[a] F statistic calculated for linear probability regression models for the outcome of program unionization using regional public sector unionization as an instrument and incorporating other covariates in the model. Cluster-robust test statistics were used for resident-level models.

© 2021 Brajcich BC et al. *JAMA Network Open*.

eTable 2. Characteristics of Survey Respondents and Nonrespondents

Characteristic	Respondents (n = 6661)	Non-Respondents (n = 1120)
Gender, No. (%)		
Male	3785 (56.8)	614 (54.8)
Female	2692 (40.4)	440 (39.3)
Missing	184 (2.8)	66 (5.9)
PGY level, No. (%)		
1	1729 (26.0)	423 (37.8)
2-3	2633 (39.5)	454 (40.5)
4-5	2299 (34.5)	243 (21.7)
Program type, No. (%)		
Academic	3955 (59.4)	682 (60.9)
Community	2706 (40.6)	438 (39.1)
Geographic region, No. (%)		
Northeast	2002 (30.1)	321 (28.7)
South	2067 (31.0)	370 (33.0)
Midwest	1587 (23.8)	265 (23.7)
West	1005 (15.1)	164 (14.6)
Unionized program, No. (%)	825 (12.4)	139 (12.4)
Urban-rural classification, No. (%)		
Large metropolitan core	3785 (56.8)	673 (60.1)
Large metropolitan fringe	832 (12.5)	119 (10.6)
Small or medium metropolitan area	2044 (30.7)	328 (29.3)
Program size, no. of examinees, median (IQR)	34 (23-49)	37 (24-51)
ABSITE score, mean (SD)	69.5 (9.6)	66.1 (10.2)
Abbreviations: ABSITE, American Board of Surgery In-Training Examination; PGY, post-graduate year		

© 2021 Brajcich BC et al. *JAMA Network Open*.

eTable 3. Resident Outcomes for Trainees at Programs That Have Been Unionized for 3 or More Years[a]

Outcome	Unionized (n = 569 residents; 26 programs)	Non-Unionized (n = 5132 residents; 259 programs)	Logistic Regression, OR (95% CI)[b]
Burnout, No. (%)	235 (41.3)	2237 (43.6)	0.93 (0.75-1.16)
Suicidal ideation, No. (%)	19 (3.3)	241 (4.7)	0.64 (0.39-1.04)
Job satisfaction			
Thoughts of attrition, No. (%)	70 (12.3)	597 (11.7)	0.97 (0.75-1.26)
Dissatisfied with decision to become a surgeon, No. (%)	31 (5.5)	259 (5.1)	1.07 (0.70-1.64)
Dissatisfied with time for rest, No. (%)	117 (20.6)	932 (18.2)	0.93 (0.66-1.32)
Duty hour violations, No. (%)	236 (42.8)	2142 (42.6)	0.86 (0.62-1.21)
Mistreatment			
Any discrimination, No. (%)[c]	274 (52.5)	2560 (53.7)	0.82 (0.64-1.04)
Bullying, No. (%)	368 (67.0)	3367 (67.0)	0.92 (0.74-1.15)
Sexual harassment, No. (%)	137 (24.9)	1558 (31.3)	0.72 (0.56-0.92)
Educational environment			
Dissatisfied with educational quality, No. (%)	88 (15.5)	473 (9.2)	1.50 (1.02-2.20)
Inadequate time for patient care, No. (%)	78 (13.8)	479 (9.4)	1.27 (0.92-1.76)
Lack of protected educational time, No. (%)	102 (18.3)	632 (12.4)	1.55 (0.97-2.46)
Inadequate time in operating room, No. (%)	81 (14.4)	375 (7.4)	1.62 (1.01-2.60)
Inadequate autonomy in operating room, No. (%)	61 (10.9)	449 (8.9)	0.98 (0.58-1.66)
Inadequate autonomy in clinical decisions, No. (%)	29 (5.2)	215 (4.2)	1.08 (0.57-2.03)
Lack of effective support staff, No. (%)	167 (29.7)	802 (15.7)	1.94 (1.26-2.97)[d]
Program not responsive to resident concerns, No. (%)	56 (9.9)	372 (7.3)	1.15 (0.74-1.77)
Program did not take wellness seriously, No. (%)	67 (11.9)	393 (7.7)	1.39 (0.94-2.07)

[a] Each row represents a separate model assessing the association of unionization status with each outcome. Residents with missing responses for the following outcomes were excluded from the model for that outcome: thoughts of attrition (n = 16), dissatisfaction with decision to become a surgeon (n = 12), dissatisfaction with time for rest (n = 9), duty hour violations (n = 118), discrimination (n = 414), bullying (n = 127), sexual harassment (n = 165), dissatisfaction with educational quality (n = 10), inadequate time for patient care (n = 33), protected educational time (n = 64), inadequate time in operating room (n = 63), inadequate autonomy in operating room (n = 64), inadequate autonomy in clinical decisions (n = 47), lack of effective support staff (n = 34), program non-responsiveness to resident concerns (n = 51), and program did not take wellness seriously (n = 37).

[b] Logistic regression models estimated odds ratio for outcome among residents at programs unionized for more than 3 years compared with programs that are not unionized or have been unionized for less than 3 years. Covariates included gender, race, Hispanic ethnicity, relationship status, parental status, census region, urban-rural classification, and program size. Odds ratios greater than 1 indicate worse outcome at unionized programs.

[c] Includes discrimination based on gender, gender identity, sexual orientation, race, ethnicity, and religion.

[d] Remains significant after Bonferroni adjustment for multiple comparisons

© 2021 Brajcich BC et al. *JAMA Network Open*.

eTable 4. Residency Program Outcomes for Programs That Have Been Unionized for 3 or More Years[a]

Outcome	Unionized (n = 25)	Non-Unionized (n = 252)	Linear Regression, mean difference (95% CI)[b]
Salary, mean (SD), $	61 625 (3903)	57 894 (4760)	205 (-1430 to 1841)
**Outcome**	**Unionized (n = 25)**	**Non-Unionized (n = 174)**	**Logistic Regression, OR (95% CI)[c]**
Vacation length, No. (%)			
Less than 4 weeks	23 (92)	56 (32)	14.14 (2.95-67.84)[d]
4 weeks	2 (8)	118 (68)	
Subsidized childcare, No. (%)	0 (0)	16 (9)	NE
Housing stipend, No. (%)	8 (32)	12 (7)	1.85 (0.50-6.83)
Relocation stipend, No. (%)	0 (0)	22 (13)	NE
Technology stipend, No. (%)	11 (44)	78 (45)	0.78 (0.30-2.01)

Abbreviations: NE, not estimable

[a] Each row represents a separate model assessing the association of unionization status with each outcome. Nine programs without available salary data were excluded, resulting in 277 programs available for analysis. For benefits, only programs for which data were available through the AMA FREIDA database or a publicly available union contract were included, resulting in 199 programs available for analysis.

[b] Linear regression model of salary for programs which have been unionized for more than 3 years compared with programs which are not unionized (n = 170) or have been unionized for 3 or fewer years (n = 4) Covariates included program size, program type, census region, urban-rural classification, and county median household income as covariates.

[c] Logistic regression models estimated odds ratio for residents at programs which have been unionized for more than 3 years compared with programs which are not unionized (n = 170) or have been unionized for 3 or fewer years (n = 4). Covariates included program size, program type, and urban-rural classification as covariates. For the housing stipend model, county-level median rent was also included.

[d] Remains significant after Bonferroni adjustment for multiple comparisons.

© 2021 Brajcich BC et al. *JAMA Network Open*.

eTable 5. Comparison of the Association Between Unionization Status and Outcome Using Linear Probability Models[a]

Outcome	Naïve Linear Probability Model, difference in probability (95% CI)	IV Linear Probability Model, difference in probability (95% CI)	Absolute Difference†	Relative Difference, %[b]
Burnout	-0.02 (-0.07 to 0.03)	0.15 (-0.11 to 0.42)	0.17	-850%
Suicidal ideation	-0.02 (-0.03 to 0.00)	-0.08 (-0.17 to 0.01)	0.10	-1000%
Job satisfaction				
Thoughts of attrition	0.00 (-0.03 to 0.03)	0.08 (-0.09 to 0.24)	0.08	NE
Dissatisfied with decision to become a surgeon	0.00 (-0.02 to 0.02)	0.11 (0.00 to 0.23)	0.11	NE
Dissatisfied with time for rest	0.00 (-0.05 to 0.06)	-0.07 (-0.28 to 0.13)	-0.07	NE
Duty hour violations	-0.03 (-0.10 to 0.04)	-0.30 (-0.65 to 0.05)	-0.27	900%
Mistreatment				
Any discrimination[c]	-0.02 (-0.06 to 0.02)	-0.05 (-0.23 to 0.13)	-0.03	150%
Bullying	-0.03 (-0.08 to 0.01)	0.05 (-0.23 to 0.33)	0.08	-267%
Sexual harassment	-0.07 (-0.11 to -0.03)[d]	-0.07 (-0.27 to 0.12)	0.00	0%
Educational environment				
Dissatisfied with educational quality	0.05 (0.00 to 0.09)	0.05 (-0.14 to 0.24)	0.00	0%
Inadequate time for patient care	0.03 (0.00 to 0.07)	-0.02 (-0.17 to 0.12)	-0.05	-167%
Lack of protected educational time	0.05 (-0.01 to 0.11)	0.00 (-0.23 to 0.23)	-0.05	-100%
Inadequate time in operating room	0.04 (-0.01 to 0.09)	0.00 (-0.15 to 0.16)	-0.04	-100%
Inadequate autonomy in operating room	0.01 (-0.03 to 0.06)	0.00 (-0.20 to 0.20)	-0.01	-100%
Inadequate autonomy in clinical decisions	0.01 (-0.02 to 0.04)	-0.03 (-0.14 to 0.09)	-0.04	-400%
Lack of effective support staff	0.09 (0.01 to 0.16)	-0.01 (-0.22 to 0.21)	-0.10	-111%
Program not responsive to resident concerns	0.02 (-0.02 to 0.05)	0.03 (-0.11 to 0.16)	0.01	50%
Program did not take wellness seriously	0.04 (0.00 to 0.07)	0.01 (-0.13 to 0.15)	-0.03	-75%
Benefits				
Vacation length	0.55 (0.37 to 0.72)[d]	0.77 (0.09 to 1.45)	0.22	40%
Subsidized childcare	-0.08 (-0.19 to 0.03)	-0.07 (-0.50 to 0.37)	0.01	-13%
Housing stipend	0.27 (0.15 to 0.39)	0.62 (0.04 to 1.20)	0.35	130%
Relocation stipend	0.04 (-0.09 to 0.16)	0.24 (-0.26 to 0.74)	0.20	500%
Technology stipend	-0.03 (-0.23 to 0.18)	-0.60 (-1.46 to 0.26)	-0.57	1900%

Abbreviations: NE, not estimable

[a] Each outcome is assessed as using a linear probability model to permit comparison of coefficients between naïve and IV models. CI denotes confidence interval.

[b] Absolute and relative difference reflects difference in estimates from IV regression models versus naïve models. NE denotes not able to be estimated (due to a naïve model estimate of 0).

[c] Includes discrimination based on gender, gender identity, sexual orientation, race, ethnicity, and religion.

[d] Remains significant after Bonferroni correction for multiple comparisons.

© 2021 Brajcich BC et al. *JAMA Network Open*.
